# A new species of the genus *Tylototriton* (Urodela, Salamandridae) from western Thailand

**DOI:** 10.3897/zookeys.1072.75320

**Published:** 2021-11-19

**Authors:** Porrawee Pomchote, Parada Peerachidacho, Axel Hernandez, Pitak Sapewisut, Wichase Khonsue, Panupong Thammachoti, Kanto Nishikawa

**Affiliations:** 1 Department of Biology, Faculty of Science, Chulalongkorn University, Bangkok 10330, Thailand; 2 Department of Environmental Sciences, Faculty of Sciences and Technics, University Pasquale Paoli of Corsica, Corte 20250, France; 3 Laboratory for Amphibian Systematic and Evolutionary Research, College of Biology and Environment, Nanjing Forestry University, Nanjing 210000, China; 4 Department of Biology, Faculty of Science, Chiang Mai University, Chiang Mai 50200, Thailand; 5 Graduate School of Global Environmental Studies, Kyoto University, Kyoto 606–8501, Japan; 6 Graduate School of Human and Environmental Studies, Kyoto University, Kyoto 606–8501, Japan

**Keywords:** conservation, crocodile newt, cryptic species, South-east Asia, taxonomy

## Abstract

We describe a new species of the newt genus *Tylototriton* from Umphang Wildlife Sanctuary, Tak Province, western Thailand based on molecular and morphological evidence and named here as *Tylototritonumphangensis***sp. nov.** The new species is assigned to the subgenus Tylototriton and differs from other species in having dark-brown to blackish-brown body and limbs, truncate snout, prominent antero-medial ends of the expansion of the dentary bones, laterally protruding quadrate regions, indistinct and small rib nodules, a well-segmented vertebral ridge, and rough dorsolateral bony ridges, which are steeper anterior, and curved medially at the posterior ends. The molecular data show that *Tylototritonumphangensis***sp. nov.** differs from *T.uyenoi* sensu stricto by a 5% genetic sequence divergence of the mitochondrial NADH dehydrogenase subunit 2 region gene. The new species and *T.uyenoi* are both endemic to Thailand, distributed along the Northwest Thai (Dawna) Uplands of Indochina. To clarify the species boundary between *Tylototritonumphangensis***sp. nov.** and *T.uyenoi*, additional field research is needed in adjacent areas. *Tylototritonumphangensis***sp. nov.** is restricted to evergreen hill forests in Umphang Wildlife Sanctuary. We suggest that the new species should be classified as Endangered (EN) in the IUCN Red List.

## Introduction

The salamandrid genus *Tylototriton* Anderson, 1871, commonly known as crocodile newts, includes 32 nominal species ranging across eastern Himalaya, eastern Nepal, northern India, Bhutan, Myanmar, central to southern China (including Hainan island), and southwards through Laos to Thailand and Vietnam (Hernandez 2016; Bernardes et al. 2020; Pomchote et al. 2020a, 2020b; Poyarkov et al. 2021a). Outside of breeding season, all known species are terrestrial and micro-endemic, regarded mostly as niche specialists that are generally found at middle to high elevations in subtropical, moist, forested environments. These provide a relatively narrow thermal range (15−24.0 °C) with a high annual rainfall, especially during the monsoon season, which ensures favorable breeding conditions and survival of *Tylototriton* species (Hernandez et al. 2018).

The genus is subdivided into three subgenera, *Tylototriton*, *Yaotriton*, and *Liangshantriton* (e.g., Dubois and Raffaëlli 2009; Fei et al. 2012; Nishikawa et al. 2013a, 2013b; Phimmachak et al. 2015; Wang et al. 2018; Poyarkov et al. 2021a) and includes several, as yet, unnamed taxa, which contain cryptic species that are morphologically difficult to distinguish (Hernandez 2016; Poyarkov et al. 2021a). Recent studies have provided a better understanding of the ecology, biology, taxonomy, phylogenetic relationships, and conservation of these endangered species that have been highly harvested in recent years throughout South-east Asia (Phimmachak et al. 2015; Hernandez et al. 2018; Wang et al. 2018; Bernardes et al. 2020; Pomchote et al. 2020a, 2020b; Poyarkov et al. 2021a). Several recent phylogenetic studies have also revealed the presence of undescribed cryptic lineages, which actually might represent independent species, in South-east Asia, especially in the Indochina region (Wang et al. 2018; Zaw et al. 2019; Bernardes et al. 2020; Pomchote et al. 2020a, 2020b; Poyarkov et al. 2021a).

To our knowledge, Thailand contains five *Tylototriton* species (Nishikawa et al. 2013a; Le et al. 2015; Pomchote et al. 2020a, 2020b). They are distributed allopatrically in high mountainous areas at altitudes above 1,000 m mean sea level throughout the northern (*T.verrucosus*, *T.uyenoi*, *T.anguliceps*, and *T.phukhaensis*), northeastern (*T.panhai*), and western (*T.uyenoi*) regions (Hernandez and Pomchote 2020a, 2020b, 2020c; Pomchote et al. 2020a, 2020b). Recent field surveys recorded several new *Tylototriton* populations distributed in the western region of Thailand, where the southernmost record in Asia of the genus was recorded (Hernandez and Pomchote 2020c). These populations were previously identified as *T.uyenoi* according to their morphological appearance and distribution, and they range from the Daen Lao and Thanon Thong Chai Ranges, southwards to the Dawna Range (Hernandez 2016, 2017; Hernandez et al. 2019).

However, according to Hernandez (2016), these newly found *T.uyenoi* populations show different phenotypes and an allopatric distribution in scattered and separated mountainous areas, resulting in the recent description of *T.phukhaensis* by Pomchote et al. (2020b). Moreover, the limited number of specimens examined in previous studies (Hernandez 2016, 2017; Hernandez et al. 2019; Hernandez and Pomchote 2020c), lack any detailed morphological examination and molecular analysis leading to the question of the taxonomic status of these populations.

As polymorphic species provide an opportunity to examine the role of isolation in populations that may contribute to the process of divergence, we assessed the western populations of *Tylototriton* species in the Umphang Wildlife Sanctuary (UPWS), Tak Province, which is located through the Dawna Range in western Thailand. This divergent population was discovered several years ago (Hernandez 2016; Hernandez et al. 2019). The molecular and morphological evidence indicate that the crocodile newt specimens from UPWS belong to a lineage distinct from the known *Tylototriton* species. As a consequence, we describe the UPWS newts as a new species, *Tylototritonumphangensis***sp. nov.**, and discuss its taxonomic relationships, distribution, and implications for conservation.

## Materials and methods

### Sampling

**Figure 1.**
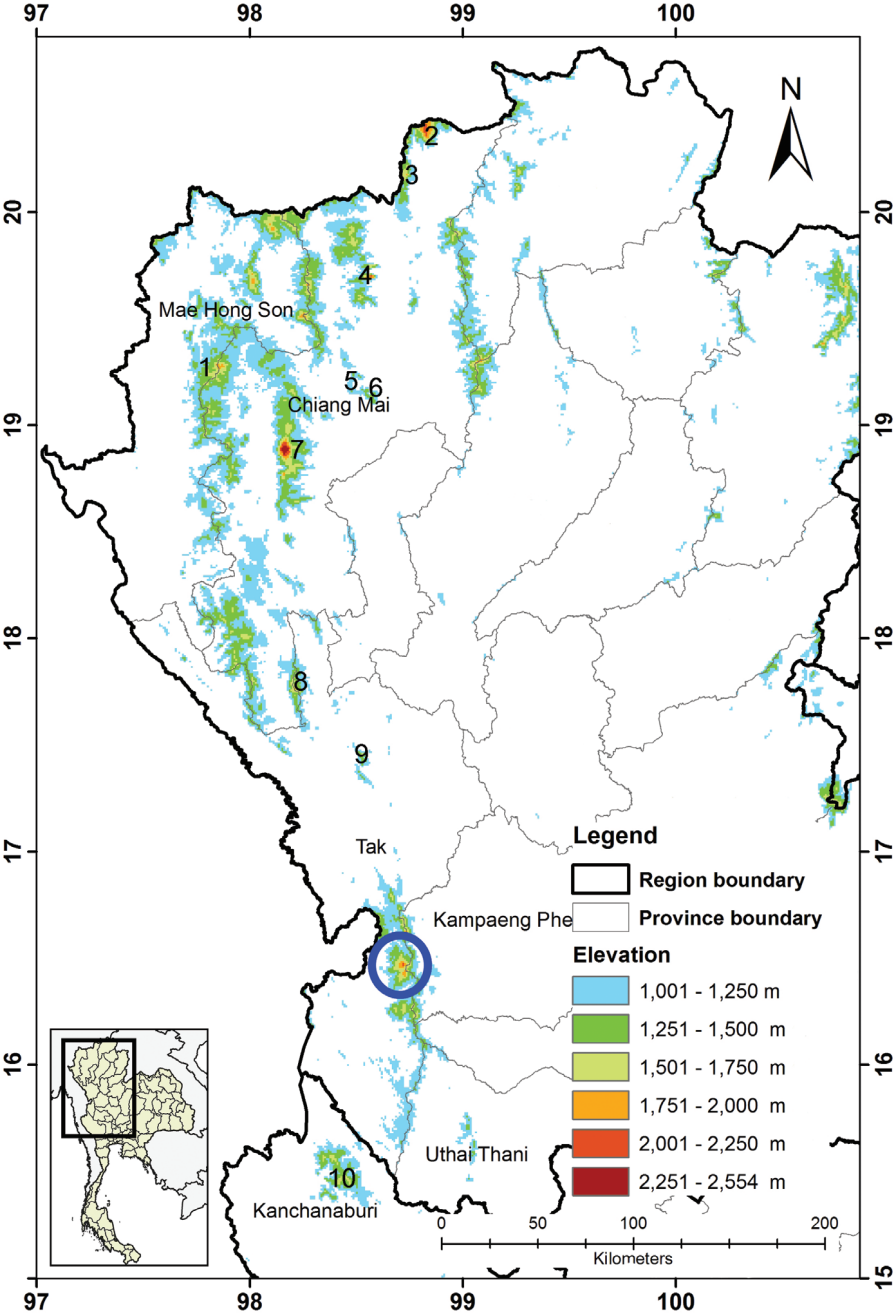
Localities for the *Tylototritonumphangensis* sp. nov., (**circle** = type locality) at Umphang Wildlife Sanctuary, Tak Province and the distribution of its closely related species. *T.uyenoi* (number) in Thailand: **1** Namtok Mae Surin NP, Mae Hong Son Province **2** Doi Mak Lang **3** Doi Ang Khang **4** Chiang Dao WS **5** Doi Suthep-Pui **6** Doi Chang Kien **7** Doi Inthanon **8** Doi Mon Jong, Chiang Mai Province **9** Doi Soi Malai, Tak Province and **10** Khao Laem NP, Kanchanaburi Province. NP = National Park and WS = Wildlife Sanctuary. The map is modified by N. Taewcharoen.

The field survey was performed on 19 June 2021 at UPWS, Tak Province, western Thailand (Fig. 1) using the visual encounter survey method (Heyer et al. 1994). Four specimens were found in a small pond that drains into a stream. The pond was surrounded by hill evergreen forest and located on the mountain at an elevation of approximately 1,150 m a.m.s.l. (above mean sea level). Specimens of *Tylototriton* were caught by hand and kept in plastic boxes for examination. The biological and physical parameters of their habitats were recorded.

All four newts were checked for sex and maturity using the cloacal characters (Pomchote et al. 2008) and were subsequently sexed as breeding males. All specimens were used for molecular and morphological analyses.

Live specimens were anesthetized by immersion in a solution of tricaine methane sulfonate (MS-222; 5 g/L) for about 5 min (Pomchote et al. 2020a), euthanized by a solution of chloretone (Heyer et al. 1994), and then measured for morphometrics and body weight (BW), as detailed below. The tissue samples (liver) of each individual were taken, and then stored in 95% (v/v) ethanol for molecular study prior to preservation. The voucher specimens were subsequently preserved in 70% (v/v) ethanol and deposited at the Chulalongkorn University Museum of Natural History (**CUMZ**).

### Molecular analyses

Total DNA was extracted from the liver using a PureDireX^TM^ genomic isolation kit (Bio-Helix, Taiwan). The mitochondrial NADH dehydrogenase 2 gene (ND2) was amplified using the polymerase chain reaction (PCR) with the SL-1 (5'–ATAGAGGTTCAAACCCTCTC–3') and the SL-2 (5'–TTAAAGTGTCTGGGTTGC ATTCA G–3') primers (Wang et al. 2018). Each PCR reaction consisted of 15 µL of OnePCR^TM^ Ultra (GeneDirex, Taiwan), which is a premixed solution, 1.5 µL of each primer (10 µM), 9 µL of UltraPure^TM^ DNase/RNase-Free distilled water (Invitrogen, USA), and 3 µL of DNA template. The thermal cycling was performed at 94 °C for 4 min, followed by 35 cycles of 94 °C for 30 s, 55 °C for 1 min, and 72 °C for 90 s (Wang et al. 2018). The PCR products were checked by agarose gel electrophoresis to confirm their size and estimate the concentration. The desired PCR products were purified and commercially sequenced by Bioneer Inc. in South Korea.

We combined the four new sequences of the UPWS samples obtained in this study with those of the other related species available from GenBank (Table 1). The optimum substitution models were selected using Kakusan 4 (Tanabe 2011). We then constructed phylogenetic trees by Bayesian inference (BI) and maximum likelihood (ML) analyses using MrBayes v. 3.1.2 (Huelsenbeck and Ronquist 2001) and RAxML v. 8 (Stamatakis 2014), respectively. The criterion used for model selection was AIC, with the codon-equal-rate model with the general time reversible model (GTR) + Gamma (G) being selected for ML and the codon-proportional model with the Hasegawa-Kishino-Yano-1985 (HKY85) model + G for each codon position for the BI. The BI analysis was performed as two independent runs of four Markov chains for 10 million generations, sampling one tree every 100 generations and calculating a consensus topology for 70,000 trees after discarding the first 30,001 trees (burn-in = 3,000,000). For the BI, we considered posterior probabilities (bpp) of 95% or greater as significant support (Leaché and Reeder 2002). The robustness of the ML tree was tested using bootstrap analysis (Felsenstein 1985) with 2,000 replicates, and we accepted tree topologies with bootstrap values (bs) of ≥ 70% to be significantly supported (Huelsenbeck and Hillis 1993). Pairwise comparisons of uncorrected sequence divergences (p-distance by 1,013 base pairs; bp) were calculated using MEGA v. 7 (Kumar et al. 2016).

**Table 1.** Specimens of *Tylototriton* and other related species used for the molecular analyses. CAS = California Academy of Sciences; CIB = Chengdu Institute of Biology; CUMZ (A) = Natural History Museum of Chulalongkorn University Section Amphibians; KIZ = Kunming Institute of Zoology; KUHE = Graduate School of Human and Environmental Studies, Kyoto University; MVZ = Museum of Vertebrate Zoology, University of California, Berkeley; NMNS = National Museum of Natural Science, Taiwan; VNMN = Vietnam National Museum of Nature; ZMMU = Zoological Museum of Moscow State University. *Topotype.

Sample no.	Species	Voucher no.	Locality	GenBank no.	Source
1	*Tylototritonumphangensis* sp. nov.	CUMZ-A-8243	Umphang Wildlife Sanctuary, Tak, Thailand	OK092618	This study
2	*Tylototritonumphangensis* sp. nov.	CUMZ-A-8244	Umphang Wildlife Sanctuary, Tak, Thailand	OK092619	This study
3	*Tylototritonumphangensis* sp. nov.	CUMZ-A-8245	Umphang Wildlife Sanctuary, Tak, Thailand	OK092620	This study
4	*Tylototritonumphangensis* sp. nov.	CUMZ-A-8246	Umphang Wildlife Sanctuary, Tak, Thailand	OK092621	This study
5	Tylototritonuyenoi*	KUHE 19147	Doi Suthep, Chiang Mai, Thailand	AB830733	Nishikawa et al. (2013a)
6	Tylototritonphukhaensis*	CUMZ-A-7719	Doi Phu Kha National Park, Nan, Thailand	MN912575	Pomchote et al. (2020b)
7	Tylototritonanguliceps*	VNMN A.2014.3	Muong Nhe, Dien Bien, Vietnam	LC017832	Le et al. (2015)
8	Tylototritonverrucosus*	KIZ 201306055	Husa, Yunnan, China	AB922818	Nishikawa et al. (2014)
9	Tylototritonpanhai*	No voucher	Phu Luang Wildlife Sanctuary, Loei, Thailand	AB830736	Nishikawa et al. (2013a)
10	Tylototritonshanjing*	NMNS 3682	Jingdong, Yunnan, China	AB830721	Nishikawa et al. (2013a)
11	*Tylototritonpulcherrimus*	KUHE 46406	Yunnan, China	AB830738	Nishikawa et al. (2013a)
12	*Tylototritonpodichthys*	KUHE 34399	Xam Neua, Houa Phan, Laos	AB830727	Nishikawa et al. (2013a)
13	Tylototritonpanwaensis*	CAS 245418	Panwa, Myitkyina, Myanmar	KT304279	Grismer et al. (2018)
14	*Tylototritonyangi*	KUHE 42282	Yunnan, China	AB769546	Nishikawa et al. (2013b)
15	Tylototritonshanorum*	CAS 230940	Taunggyi, Shan, Myanmar	AB922823	Nishikawa et al. (2014)
16	*Tylototritonhimalayanus*	MVZ no number	Nepal	DQ517854	Weisrock et al. (2006)
17	Tylototritonkachinorum*	ZMMU A5953	Indawgyi, Kachin, Myanmar	MK097273	Zaw et al. (2019)
18	*Tylototritonkweichowensis*	MVZ 230371	Daguan, Yunnan, China	DQ517851	Weisrock et al. (2006)
19	*Tylototritontaliangensis*	KUHE 43361	Unknown, China	AB769543	Nishikawa et al. (2013b)
20	Echinotritonandersoni*	KUHE no number	Nago, Okinawa, Japan	AB769545	Nishikawa et al. (2013b)

### Morphological examination

The morphometric characters of the UPWS newts were compared with those of *T.uyenoi* because their appearances and color pattern are rather similar; moreover, previous studies identified the UPWS newts as *T.uyenoi* (Hernandez et al. 2019). Note that the other four *Tylototriton* species from Thailand (*T.verrucosus*, *T.anguliceps*, *T.phukhaensis*, and *T.panhai*) were not included in this morphometric study for two reasons. Firstly, the external morphology of *T.verrucosus*, *T.anguliceps*, and *T.phukhaensis* was clearly different from that of *T.uyenoi* (see Pomchote et al. 2020a, 2020b), although morphological comparisons using the published literature were made (see comparisons below). Secondly, *T.panhai* has different color pattern from that of the other Thai *Tylototriton* species; moreover, *T.panhai* is a member of another lineage, the subgenus Yaotriton (Nishikawa et al. 2013a).

We compared the reported morphometrics of a total of 12 specimens, the four *Tylototriton* sp. from UPWS (four males: CUMZ-A-8243 to -8246) of this study, and eight specimens of *T.uyenoi* obtained previously from the same localities of the holotype and paratypes of *T.uyenoi* (Nishikawa et al. 2013a). The specimens of *T.uyenoi* were loaned from the Natural History Museum, National Science Museum, Thailand (**THNHM**): topotypic specimens THNHM 10319–10320, 20170 (three males) from Doi (= Mountain in Thai language) Suthep-Doi Pui National Park (NP), Chiang Mai Province; and THNHM 13866, 13868, 13870–13871 (four males), and THNHM 13869 (one female) from Doi Inthanon NP, Chiang Mai Province, which is the same locality as the paratypes.

The following 27 measurements were taken for morphometric comparisons, where the character definitions are given in Nishikawa et al. (2011): **SVL** (snout–vent length); **HL** (head length); **HW** (head width); **MXHW** (maximum head width); **SL** (snout length); **LJL** (lower jaw length); **ENL** (eyelid-nostril length); **IND** (internarial distance); **IOD** (interorbital distance); **UEW** (upper eyelid width); **UEL** (upper eyelid length); **OL** (orbit length); **AGD** (axilla-groin distance); **TRL** (trunk length); **TAL** (tail length); **VL** (vent length); **BTAW** (basal tail width); **MTAW** (medial tail width); **BTAH** (basal tail height); **MXTAH** (maximum tail height); **MTAH** (medial tail height); **FLL** (forelimb length); **HLL** (hindlimb length); **2FL** (second finger length); **3FL** (third finger length); **3TL** (third toe length); and **5TL** (fifth toe length). All measurements were taken using a digital sliding caliper to the nearest 0.01 mm, subsequently rounded to 0.1 mm. Each measurement was taken three times and the average was used for further analyses. Their body weights (**BW**) were recorded using a digital weighing scale to the nearest 0.1 gm.

For morphological comparisons, the data for the other related species were taken from the related literatures (Fang and Chang 1932; Liu 1950; Nussbaum et al. 1995; Böhme et al. 2005; Hou et al. 2012; Nishikawa et al. 2013a, 2014; Khatiwada et al. 2015; Le et al. 2015; Phimmachak et al. 2015; Grismer et al. 2018, 2019; Zaw et al. 2019; Pomchote et al. 2020a, 2020b).

### Statistical analysis

We compared the SVL, BW, and the other 25 ratio values to SVL (presented as % SVL) between *Tylototriton* sp. from UPWS and the other *T.uyenoi* specimens. Differences in morphological characters between the *Tylototriton* sp. from UPWS and *T.uyenoi* were analyzed by the Mann-Whitney *U* test. The relationships of all morphometric characters were examined using principal component analysis (PCA). Note that the vent length of the one *T.uyenoi* female (THNHM 13869) was excluded from the morphological comparison because this parameter is much longer in males than in females [RVL 7.4 vs 1.7 and 1.9; 9.3 vs 4.0 in *T.uyenoi*, data from Nishikawa et al. (2014) and the present study, respectively]. All statistical analyses were performed using the SPSS v. 22 for Windows. Statistical significance was accepted at the p < 0.05 level.

## Results

### Molecular analyses

We obtained 452–1,039 bp sequences of the partial ND2 region for 20 specimens, including the outgroup (Table 1). The sequences of the four specimens from UPWS (this study) were the same, and of the 1,039 nucleotide sites, 340 were variable and 158 were parsimony informative within the ingroup (sequence statistics available upon request from the senior author). The mean likelihood score of the BI analyses for all trees sampled at stationary was −4033.667. The likelihood value of the ML tree was −3955.266.

**Figure 2.**
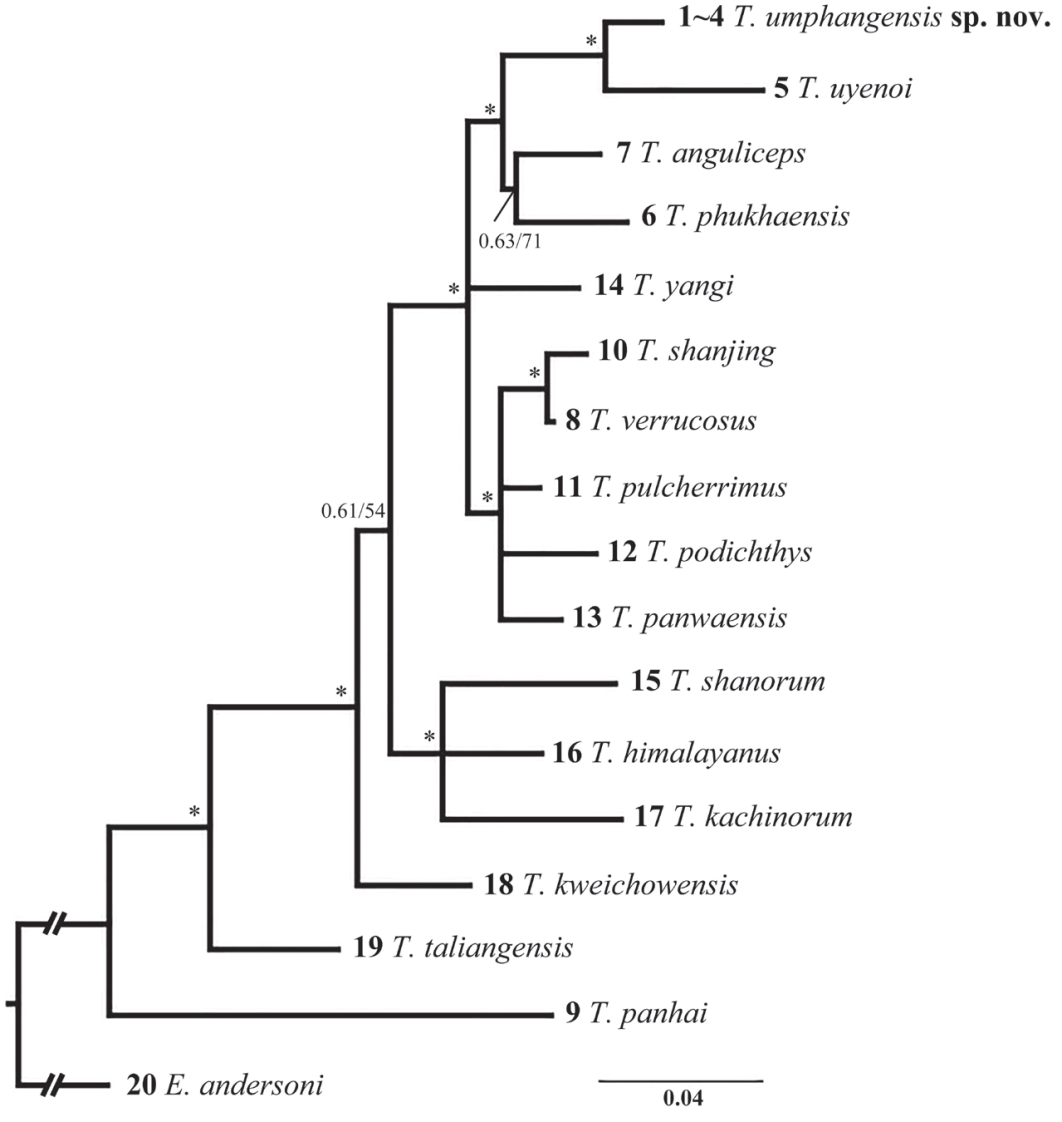
Bayesian inference tree based on the partial ND2 gene for the samples examined. Numbers above branches represent the bpp/bs, and asterisks indicate nodes with bpp ≥ 0.95 and bs ≥ 70%. Numbers at branches tips are the sample numbers, as shown in Table 1. Scale bar = 0.04 substitutions/site.

Phylogenetic analyses employing the BI and ML criteria yielded nearly identical topologies and so we present only the BI tree in Figure 2. Monophyly of the subgenus Tylototriton (all samples except Samples 9 and 20) was fully supported in the BI and ML trees (bpp = 98% and bs = 96%). Within the subgenus, *T.taliangensis* was first separated from the remaining lineages. The latter group was further divided into two clades: one including *T.shanorum*, *T.himalayanus*, and *T.kachinorum*; the other included the remaining lineages. The newts from UPWS (same sequence) were nested in the latter clade and was first clustered with *T.uyenoi* with significant support.

The *p*-distances between each pair of a total 17 haplotypes recognized above ranged from 1.4% (between *T.verrucosus* and *T.shanjing*) to 18.8% (between *Echinotriton* and *T.uyenoi* and between *Echinotriton* and *T.kachinorum*) (Table 2). The distance between the newts from UPWS and its sister species *T.uyenoi* was 5.0%, which was larger than the 24 heterospecific combinations in this study.

**Table 2.** Genetic uncorrected *p*-distance (%) of the ND2 region between samples examined in this study.

	**Species**	**Sample no.**															
		**1**	**5**	**6**	**7**	**8**	**9**	**10**	**11**	**12**	**13**	**14**	**15**	**16**	**17**	**18**	**19**
**1**	*Tylototritonumphangensis* sp. nov.																
**5**	*Tylototritonuyenoi*	0.050															
**6**	*Tylototritonphukhaensis*	0.061	0.091														
**7**	*Tylototritonanguliceps*	0.052	0.082	0.045													
**8**	*Tylototritonverrucosus*	0.039	0.073	0.057	0.057												
**9**	*Tylototritonpanhai*	0.136	0.145	0.138	0.134	0.127											
**10**	*Tylototritonshanjing*	0.043	0.073	0.061	0.057	0.014	0.132										
**11**	*Tylototritonpulcherrimus*	0.048	0.073	0.054	0.045	0.025	0.116	0.029									
**12**	*Tylototritonpodichthys*	0.063	0.098	0.068	0.057	0.041	0.127	0.054	0.039								
**13**	*Tylototritonpanwaensis*	0.043	0.082	0.059	0.045	0.020	0.122	0.034	0.018	0.029							
**14**	*Tylototritonyangi*	0.043	0.075	0.059	0.039	0.034	0.132	0.043	0.027	0.045	0.027						
**15**	*Tylototritonshanorum*	0.075	0.095	0.079	0.070	0.059	0.122	0.063	0.059	0.079	0.063	0.070					
**16**	*Tylototritonhimalayanus*	0.068	0.084	0.063	0.057	0.054	0.118	0.054	0.057	0.063	0.057	0.063	0.052				
**17**	*Tylototritonkachinorum*	0.091	0.118	0.088	0.079	0.073	0.134	0.082	0.079	0.082	0.066	0.082	0.079	0.054			
**18**	*Tylototritonkweichowensis*	0.070	0.091	0.068	0.054	0.052	0.118	0.057	0.054	0.066	0.054	0.057	0.063	0.052	0.066		
**19**	*Tylototritontaliangensis*	0.077	0.088	0.073	0.075	0.057	0.107	0.057	0.057	0.070	0.061	0.073	0.068	0.057	0.075	0.043	
**20**	*Echinotritonandersoni*	0.186	0.188	0.179	0.184	0.172	0.175	0.175	0.166	0.177	0.166	0.184	0.179	0.175	0.188	0.168	0.156

### Morphological examination

A total of 12 specimens were used for morphometric comparisons (Table 3). With respect to the SVL, the UPWS population was significantly larger than *T.uyenoi* (*p* = 0.027), but with significantly smaller RHL (*p* = 0.042), RENL (*p* = 0.007), RUEW (*p* = 0.017), RUEL (*p* = 0.007), and ROL (*p* = 0.007) % SVL measurements, and a significantly larger R2FL (*p* = 0.007) measurement than those for *T.uyenoi*.

**Table 3.** Morphometric comparisons of the examined specimens of *Tylototriton* [mean ± SD of SVL (in mm), mean ± SD of BW (g), and median of ratios of characters (R: % SVL), with range in parentheses]. Character abbreviations refer to the text.

	***T.umphangensis* sp. nov.**	***T.uyenoi***			***T.umphangensis* sp. nov.**	***T.uyenoi***	
	4 males	7 males	1 female		4 males	7 males	1 female
SVL	72.0 ± 4.4* (65.6–75.3)	62.7 ± 4.8 (55.5–67.4)	60.9	RTRL	76.8 (75.4–77.4)	75.2 (71.9–77.5)	76.2
BW	12.1 ± 1.4 (10.2–13.3)			RTAL	104.7 (91.9–107.3)	106.3 (67.4–117.0)	85.2
RHL	23.0* (22.0–25.2)	25.2 (24.1–26.1)	24.9	RVL	8.0 (7.3–9.4)	9.1 (7.8–11.5)	4.0
RHW	21.4 (19.4–22.7)	22.3 (19.9–25.4)	26.5	RBTAW	14.5 (12.6–15.1)	13.1 (12.2–15.0)	13.1
RMXHW	25.6 (25.0–26.9)	25.5 (24.6–28.6)	28.1	RMTAW	2.3 (2.2–2.4)	2.7 (1.6–3.3)	2.1
RSL	8.8 (8.2–9.8)	8.7 (7.8–9.4)	9.0	RBTAH	15.0 (11.9–15.3)	11.8 (10.0–13.8)	15.5
RLJL	22.8 (22.1–23.5)	22.9 (20.7–23.3)	23.2	RMXTAH	11.1 (8.8–12.1)	12.0 (10.3–13.8)	16.9
RENL	5.8* (5.6–6.2)	7.1 (6.2–7.4)	6.9	RMTAH	10.6 (7.9–12.0)	10.0 (8.0–13.6)	16.2
RIND	6.2 (5.8–6.5)	6.4 (6.0–7.8)	7.6	RFLL	37.0 (34.2–40.5)	39.4 (35.6–42.6)	33.1
RIOD	13.2 (12.9–13.7)	13.2 (12.4–14.3)	14.2	RHLL	38.4 (35.2–41.9)	43.6 (37.9–50.9)	40.1
RUEW	2.5* (2.3–2.9)	3.2 (2.6–3.5)	2.9	R2FL	7.1* (6.7–8.1)	5.9 (5.1–6.5)	4.7
RUEL	6.0* (5.5–6.4)	7.2 (6.5–7.8)	7.5	R3FL	7.6 (5.6–8.9)	6.6 (5.5–8.2)	6.3
ROL	3.0* (2.7–3.3)	4.6 (4.0–5.0)	4.2	R3TL	9.3 (8.9–11.0)	9.0 (8.3–12.5)	9.1
RAGD	53.7 (51.9–54.4)	52.2 (46.7–57.2)	50.8	R5TL	4.9 (4.8–5.7)	4.4 (3.5–6.5)	4.1

* *p* < 0.05 compared to *T.uyenoi* (Mann-Whitney *U* test)

**Figure 3.**
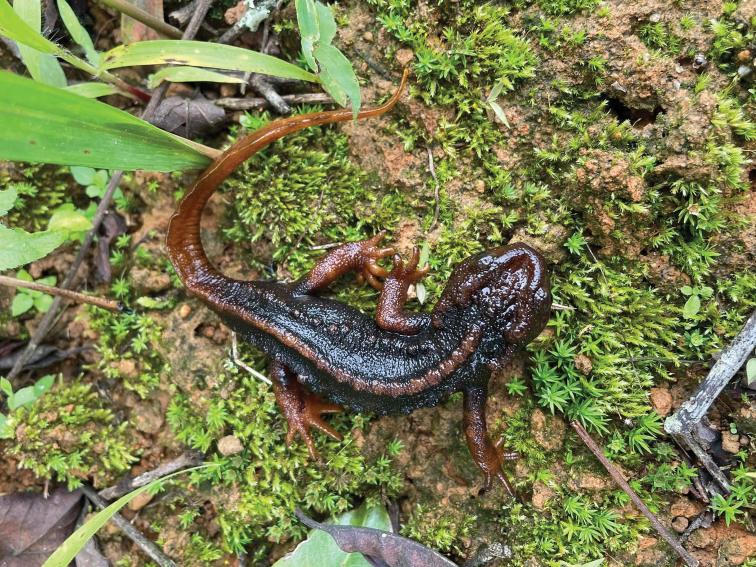
Male *Tylototritonumphangensis* sp. nov.

**Figure 4.**
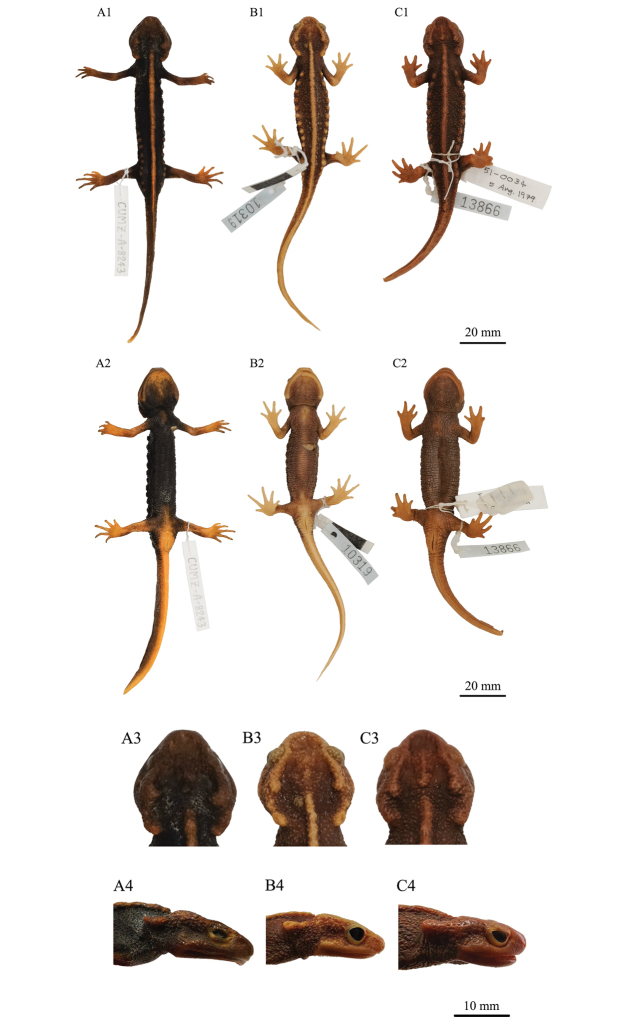
The male holotype of *Tylototritonumphangensis* sp. nov. and other specimens of *T.uyenoi* in preservative. **A** Holotype specimen of *Tylototritonumphangensis* sp. nov. (CUMZ-A-8243) **B** Topotypic specimen of *T.uyenoi* (THNHM 10319) from Doi Suthep-Doi Pui NP, Chiang Mai Province **C** specimen of *T.uyenoi* (THNHM 13866) from Doi Inthanon NP, Chiang Mai Province **A1**–**C1** dorsal view of the body. **A2**–**C2** ventral view of the body. **A3**–**C3** dorsal view of the head **A4**–**C4** lateral view of the head. Scale bars: 20 mm (**A1**–**C1, A2**–**C2**); 10 mm (**A3**–**C3, A4**–**C4**).

**Figure 5.**
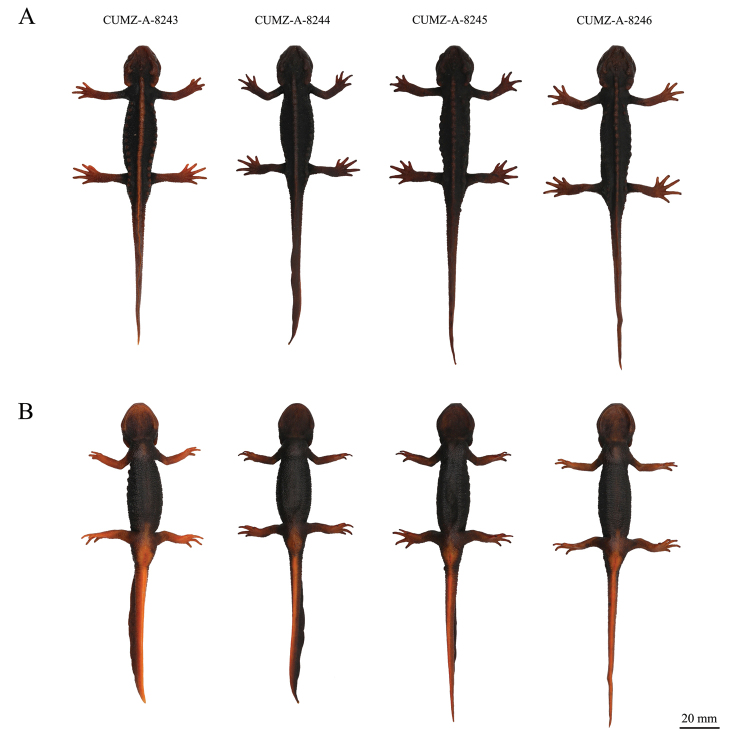
Holotype (CUMZ-A-8243) and paratypes (CUMZ-A-8244, CUMZ-A-8245, and CUMZ-A-8246) of *Tylototritonumphangensis* sp. nov. before preservation. **A** dorsal view **B** ventral view. Scale bar: 20 mm.

In life, the dorsal ground color was dark brown to black (Nishikawa et al. 2013a; Pomchote et al. 2020a), while in preservative, the background coloration of *T.uyenoi* was light brown to cream (Nishikawa et al. 2013a) or light brown to brown (this study), although the UPWS samples tended to be the blackish both in life and in preservative. In *T.uyenoi*, the dorsal and ventral head, parotoids, vertebral ridge, rib nodules, limbs, vent, and tail were orange to reddish-brown in life (Nishikawa et al. 2013a; Pomchote et al. 2020a) and light brown to orange-brown (Nishikawa et al. 2013a) or cream to orange-brown (this study) in preservative, although the UPWS samples had darker markings than *T.uyenoi*, both in life and in preservative (Figs 3–5).

The UPWS samples and *T.uyenoi* also showed a few similar morphological characteristics. For example, the sagittal ridge on head and the parotoids were distinct and projected posteriorly. However, morphological differences between the UPWS population and *T.uyenoi* were also present (Fig. 4).

The snout of the UPWS population was truncate, while that of *T.uyenoi* was almost rounded to blunt, except for two specimens (THNHM 10319 and 10320) that were relatively truncate. In lateral view, the degree that the snout projects beyond the lower jaw was more distinct in *T.uyenoi* than in the UPWS population that hardly projected beyond the lower jaw.

The dorsolateral bony ridges of the UPWS population were rough, steeper anteriorly, and curved medially at the posterior ends, while those of *T.uyenoi* were rough, especially from above the eye to above the anterior end of the parotoid, less steep anteriorly, and weakly or rather curved medially at the posterior ends.

In lateral view, the parotoids of the UPWS population were oriented rather parallel to the body axis and the posterior part curved upwards, while those of *T.uyenoi* were oriented obliquely downwards or rather parallel relative to the body axis.

In dorsal view, the quadrate regions of the UPWS population protruded laterally, while those of *T.uyenoi* were weakly curving.

In the urodeles, the dentaries are elongated, paired bones that curve medially. The left and right dentaries touch each other antero-medially on the lower jaw. At the antero-medial ends, some expansions are developed posteriorly in the dorsal view, while in the anterior view this expansion slightly develops in the ventral direction (e.g., Villa et al. 2014; Parra Olea et al. 2020; Ponssa and Abdala 2020). This expansion is prominently present in the UPWS population (Fig. A4), both in life and in preservative, whereas it was absent in *T.uyenoi* (Figs B4–C4).

The vertebral ridge of the UPWS population and *T.uyenoi* was segmented from the anterior end to the tail base but was less segmented in *T.uyenoi* than in the UPWS samples, especially the two paratypes CUMZ-A-8245 and 8246, which were prominently segmented.

The rib nodules of *T.uyenoi* were isolated, rounded, distinct but small, and forming knob-like warts, with the number of rib nodules ranging from 12–15, but almost all the specimens had 14 warts on each side of body. In contrast, the rib nodules of the UPWS newts were indistinct and small in shape, ranging from 14–15 warts.

**Figure 6.**
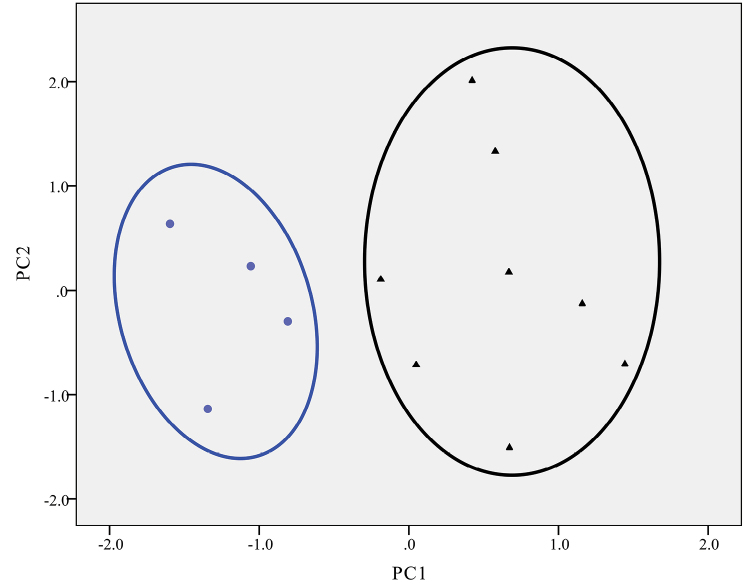
The PCA plots of PC1 vs PC2 for morphometric parameters between the UPWS population (*Tylototritonumphangensis* sp. nov. described herein) (circle) and specimens of *T.uyenoi* (triangle).

The overall morphological differences were examined using PCA for the UPWS population and *T.uyenoi*. The first two principal components (PCs) explained 49.0% of the total variation. The two-dimensional plots of PC1 vs PC2 showed that the UPWS population was clustered together and separated from *T.uyenoi* (Fig. 6).

Based on the molecular and morphological evidence, the *Tylototriton* sp. from UPWS, Tak Province, western Thailand is confirmed as an undescribed species. Therefore, we describe it as a new species, *Tylototritonumphangensis* sp. nov.

## Systematics

### Tylototriton umphangensis sp. nov.

Taxon classificationAnimaliaCaudataSalamandridae

2B42FF3F-D753-5E06-AE91-AE2800481B22

http://zoobank.org/D280A352-65C9-4F84-91BF-53F19B954E87

Thai name: Ka Tang Nam Umphang English name: Umphang crocodile newt Figures 3 , 4 , 5

T. uyenoi : (referring to the population from Umphang, Tak Province): Hernandez et al. 2019, page 18.

#### Holotype.

CUMZ-A-8243, adult male, collected from Umphang Wildlife Sanctuary, Tak Province, western Thailand, approximate coordinate 16°12’N, 98°58’E; ca 1,150 m a.m.s.l., collected on 19 June 2021 by Porrawee Pomchote and Pitak Sapewisut.

#### Paratypes.

CUMZ-A-8244, CUMZ-A-8245, and CUMZ-A-8246; three adult males, same data as the holotype.

#### Etymology.

The specific epithet *umphangensis* refers to Umphang Wildlife Sanctuary, the type locality of the new species.

#### Diagnosis.

The new species is placed in the genus *Tylototriton* by having a combination of dorsal granules present, dorsolateral bony ridges on head present, knob-like warts (rib nodules) on dorsolateral body present, and quadrate spine absent. *Tylototritonumphangensis* sp. nov. differs from its congeners by having the following morphological characters: medium-sized, adult SVL 65.6–75.3 mm in males; skin rough with fine granules; snout truncate; quadrate regions laterally protruding; antero-medial ends of dentaries distinctly expanded; dorsolateral bony ridges on head prominent, steep, rough, narrow, and posterior ends curved medially; parotoids distinct, oriented rather parallel to the body axis and posterior part curved upwards in the lateral view; vertebral ridge distinct and segmented; rib nodules 14–15, small, and indistinct; limbs long and thin; tips of forelimbs and hindlimbs overlapping when adpressed along the body; tail thin.

#### Description of holotype.

Body rather slim and long (RTRL 76.6%); skin rough; fine granules dense on dorsum, dense on sides of body and tail, and arranged in transverse striations on mid-ventrum; head longer than wide (HW/HL 0.97), hexagonal in shape, depressed, and slightly oblique in profile; snout truncate, hardly projecting beyond lower jaw; nostrils close to snout tip, not visible from dorsal view; quadrate regions protruding laterally from dorsal view; antero-medial ends of dentaries distinctly expanded; dorsolateral bony ridges on head narrow, rough, and posterior ends curved proximally; sagittal ridge on head short and weak; labial fold absent; tongue oval, attached to anterior floor of mouth, free laterally and posteriorly; vomerine tooth series in an inverted V-shape, converging anteriorly and reaching choanae; parotoids distinct, projecting posteriorly, posterior ends slightly curved medially, oriented rather parallel to body axis and curved upwards in lateral view; gular fold present; costal folds absent; vertebral ridge prominent, narrow, and slightly segmented from neck to groin, separated from sagittal ridge on head; two low and flat bony ridges on the dorsal head surface forming a “V” shape, connected with the anterior end of vertebral ridge; rib nodules small, indistinct, forming knob-like warts, 15 on each side of body from axilla to base of tail; rib nodules slightly increasing in size from most anterior to forth nodule, then decreasing posteriorly; forelimbs (34.2% SVL) shorter than hindlimbs (40.0% SVL); tips of forelimb and hindlimb overlapping when adpressed along body; fingers and toes well developed, free of webbing; fingers four, comparative finger lengths 2 > 3 > 1 > 4; toes five, comparative toe lengths 4 > 3 > 2 > 5 > 1; tail laterally compressed, dorsal fin more distinct posteriorly, ventral edge smooth, tip pointed; tail short (91.9% SVL); cloaca slightly swollen; vent slit longitudinal.

#### Color of holotype.

In life, dorsal ground coloration is dark-brown to blackish-brown, while the ventral color is slightly lighter than dorsum. Dorsal, ventral, and lateral of head, parotoids, vertebral ridge, rib nodules, limbs, vent region, and whole tail are orange-brown. Tip of tail is slightly lighter than dorsal and lateral sides of tail. Ventral side of head, part of pectoral and pubic region, limbs, and tail prominently lighter than dorsum. The lightest is the ventral edge of the tail. The lighter region between the ventral edge of the tail and the area of the vent is connected. Color of digit tips is dark brown. After a week in preservation, the color pattern is rather similar to that in life.

#### Measurement of holotype (in mm).

SVL 72.7; HL 16.4; HW 15.9; MXHW 18.1; SL 6.3; LJL 16.7; ENL 4.2; IND 4.3; IOD 9.6; UEW 1.6; UEL 4.7; OL 2.1; AGD 39.3; TRL 55.7; TAL 66.8; VL 6.3; BTAW 10.6; MTAW 1.7; BTAH 11.2; MXTAH 8.8; MTAH 8.7; FLL 24.9; HLL 29.1; 2FL 5.3; 3FL 4.1; 3TL 6.5; and 5TL 3.5.

#### Variation.

Some differences in morphology were observed among the four specimens. The dorsolateral bony ridges on the head of one paratype (CUMZ-A-8245) are rougher than the holotype and the other paratypes. The sagittal ridge on the head of one paratype (CUMZ-A-8244) is smaller and weaker than the holotype and the other paratypes. Two paratypes (CUMZ-A-8245 and CUMZ-A-8246) have a more distinctly segmented vertebral ridge than the holotype and the other paratype. The holotype has much more distinct rib nodules than the three paratypes. Sizes of rib nodules varied from rounded anteriorly to irregularly shaped posteriorly among the paratypes. One paratype (CUMZ-A-8246) has an undulated surface of the dorsal fin, while the other type specimens have an even-surfaced dorsal fin. Type specimens are generally similar in color pattern, but the coloration of the dorsal, ventral, and lateral head, parotoids, vertebral ridge, rib nodules, limbs, and whole tail is much lighter in the holotype than the three paratypes. The color of the digit tips of the holotype is dark brown, but those of the paratypes are black. Morphological variations between the specimens are shown in Figure 5.

#### Comparisons.

*Tylototritonumphangensis* sp. nov. differs from the other species of subgenus Tylototriton as follows: from *T.taliangensis* by having orange-brown markings on the head, trunk, limbs, and tail (vs uniformly black body except for distal fingers, toes, and posterior parotoids in *T.taliangensis*); from *T.kweichowensis* and *T.pseudoverrucosus* by having separated rib nodules (vs connected orange markings forming continuous dorsolateral lines in *T.kweichowensis* and *T.pseudoverrucosus*); from *T.shanorum* and *T.anguliceps* by having a sagittal ridge and rather steep dorsolateral bony ridges on the head (vs no sagittal ridge and rather flat dorsolateral bony ridges on head in *T.shanorum*, and prominent sagittal ridge and the posterior ends of dorsolateral bony ridges distinctly curved medially in *T.anguliceps*); from *T.ngarsuensis* by having truncate snout in dorsal view (vs rounded in *T.ngarsuensis*); from *T.himalayanus* by lacking grooves on either side at the basal tail (vs present in *T.himalayanus*); from *T.yangi* by having uniformly orange-brown parotoids (vs black coloration except for posterior end of parotoids with orange coloration in *T.yangi*); from *T.kachinorum*, *T.pulcherrimus*, and *T.shanjing* by having light orange-brown on part of pubic region (vs light yellowish-grey ventral surfaces in *T.kachinorum*, and yellowish-orange to bright yellow ventral trunk in *T.pulcherrimus* and *T.shanjing*); from *T.verrucosus* by having rough dorsolateral bony ridges (vs smooth in *T.verrucosus*); from *T.podichthys* and *T.phukhaensis* by having short and weak sagittal ridge on the head (vs indistinct sagittal ridge on head in *T.podichthys*, and narrow, long, and prominent sagittal ridge on head in *T.phukhaensis*); from *T.panwaensis* by having narrow vertebral ridge (vs wide in *T.panwaensis*).

#### Distribution.

Umphang Wildlife Sanctuary, Tak Province, western Thailand (Fig. 1). The Umphang Wildlife Sanctuary is located along the Dawna Range, which is a mountain range in eastern Myanmar and northwestern Thailand. Thus, this species is expected to also occur in Myanmar and elsewhere in western Thailand.

#### Natural history.

**Figure 7.**
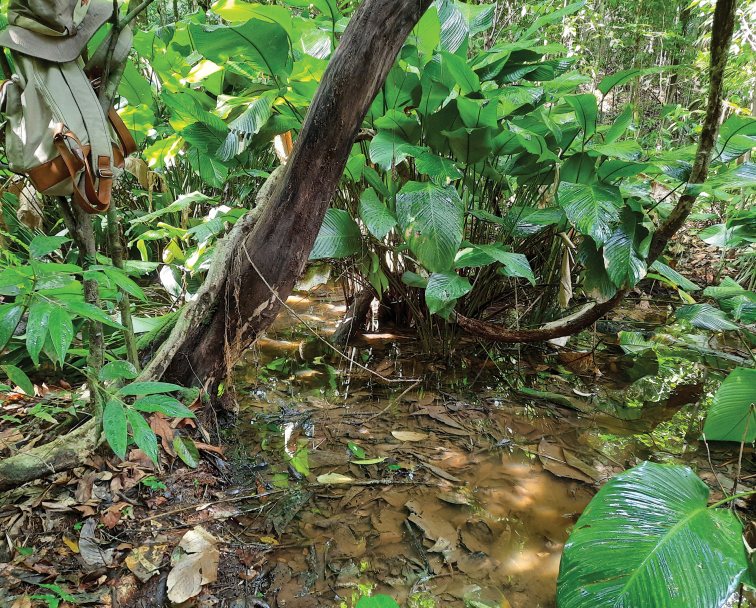
Habitat at the type locality of *Tylototritonumphangensis* sp. nov. at Umphang Wildlife Sanctuary, Tak Province, western Thailand.

All specimens were found during the afternoon at around 14:30 h hidden under leaf litter and between stems of arrowroot plants (family Marantaceae) in a small ephemeral pond (Fig. 7) during the rainy season, which is the breeding season of *Tylototriton* species. The pond had clear water and the bottom was covered with dense leaf litter. The surrounding area was composed of hill evergreen forest. The pond size was approximately 520 cm long, 270 cm wide, and 17 cm in maximum depth. The water temperature was 23.1 °C. The water quality parameters were: pH 6.4; dissolved oxygen 4.13 mg/L; conductivity 23 µS/cm; total dissolved solid 15 mg/L; and turbidity 7.6 NTU. No fish were observed.

## Discussion

Molecular and morphological evidence indicate that the newts found at UPWS, Tak Province, western Thailand are a distinct, new species described here. With our description of this new species, the number of *Tylototriton* species is now 33, with six of them present in Thailand: *T.verrucosus*, *T.uyenoi*, *T.panhai*, *T.anguliceps*, *T.phukhaensis*, and *T.umphangensis* sp. nov. Three of these six species are endemic to Thailand (*T.uyenoi*, *T.phukhaensis*, and *T.umphangensis* sp. nov.). Thus, Thailand has the third highest number of species of *Tylototriton* and the second highest in Indochina; the highest number of species is in China (17) followed by Vietnam (7) (Frost 2021).

*Tylototritonumphangensis* sp. nov. has been confused with *T.uyenoi* because of these species have morphological similarities and a similar distribution (Hernandez et al. 2019). In the present study, we compared *T.umphangensis* sp. nov. with *T.uyenoi*, the latter having been described and named based on a holotype and eight paratypes (total of nine adult males) collected from Phuping Rajanives Palace, Doi Suthep and the Royal Garden Siribhume, Doi Inthanon, Chiang Mai Province, respectively (Nishikawa et al. 2013a). According to Nishikawa et al. (2013a), *T.uyenoi* specimens are basically similar in morphology and color pattern, but show variations in the degree of segmented vertebral ridge, size of rib nodules, texture of dorsolateral bony ridges, and color markings. These morphological variations are also present in the specimens of *T.umphangensis* sp. nov. that we examined in our study. However, *T.umphangensis* sp. nov. can be distinguished from the most closely related species (*T.uyenoi*) and other congeners.

Geographic isolation may limit gene flow and promote genetic differentiation among populations which can result in the formation of new species (Eckert et al. 2008; Qian et al. 2017). Tak Province is located in the Northwest Thai (Dawna) Uplands of Indochina (Poyarkov et al. 2021b), which consists of several high-mountain areas in the three major mountain ranges: (i) Thanon Thong Chai and (ii) Daen Lao Ranges in northernmost Tak Province, and (iii) Dawna Range in most areas of Tak Province. The Thanon Thong Chai Range has *T.uyenoi* in Namtok Mae Surin NP, Mae Hong Son Province (Pomchote et al. 2020a). The Daen Lao Range has *T.uyenoi* populations in Doi Ang Khang, Doi Chang Kien, Chiang Dao Wildlife Sanctuary (WS), Doi Inthanon, Doi Mak Lang or Doi Lang, and Doi Suthep-Pui in Chiang Mai Province (Pomchote et al. 2008; Nishikawa et al. 2013a; Michaels 2014; Hernandez 2016; Hernandez et al. 2019); Doi Soi Malai, Tak Province (Hernandez 2017); and Doi Mon Jong, Chiang Mai Province (Hernandez et al. 2019). The Dawna Range supports *T.uyenoi* in Khao Laem NP in Kanchanaburi Province (Hernandez and Pomchote 2020c) and Umphang, Tak Province (Hernandez et al. 2019; this study) (Fig. 1).

Hernandez et al. (2018) stated that *Tylototriton* species are niche specialists because they reside at high elevations with moist, and cool conditions, a narrow thermal range, and high rainfall during the breeding season. This is consistent with previous studies that Thai *Tylototriton* species are distributed in high mountainous areas at an altitude of more than 1,000 m a.m.s.l. (Pomchote et al. 2008, 2020a, 2020b). Following to previous studies (Pomchote et al. 2008, 2020a, 2020b), we defined lowland and highland areas according to the distribution of six *Tylototriton* species in Thailand: lowlands are areas below 1,000 m a.m.s.l. and uplands are areas above 1,000 m a.m.s.l. Thus, the lowland areas, located between each highland area, of the Northwest Thai Uplands may serve as a barrier restricting the gene flow between *Tylototriton* populations. Consequently, further morphological and molecular analyses, as well as field surveys in Tak Province and its nearby areas located along Thanon Thong Chai, Daen Lao, and Dawna Ranges, need to be done to clarify the species boundary between *T.umphangensis* sp. nov. and *T.uyenoi*.

According to previous data (Watchara Sanguansombat and Chattraphas Pongcharoen, personal communication) and the check list of fauna diversity of UPWS (Department of National Parks, Wildlife and Plant Conservation), *T.verrucosus* (now named *T.umphangensis* sp. nov.) were first found near an artificial pond adjacent to a deserted hut near a road that was about 6 km from the Mae Klong Khi Forest Ranger Station. Our field survey was conducted there on 18 June 2021 at night, but we did not find any newts. Not only did this pond have fish (released by someone?), but this area is also under construction. Moreover, there are cattle that belong to local people roaming freely in UPWS that may cause damage to the forest, including breeding sites of the newts, as previously reported in other NPs, such as in Phu Suan Sai NP, Loei Province that harbors *T.panhai* (Hernandez and Pomchote 2020a); and in Doi Phu Kha NP, Nan Province that harbors *T.phukhaensis* (Pomchote et al. 2020b). Thus, effects from anthropogenic activities, including cattle, should be evaluated in detail. *Tylototritonumphangensis* sp. nov. is currently only known from the hill evergreen forests of UPWS. We suggest that the new species should be classified as Endangered (EN) in the IUCN Red List and that it needs further conservation management.

## Supplementary Material

XML Treatment for Tylototriton umphangensis
